# A comparison between size of the occluder device and two-dimensional transoesophageal echocardiographic sizing of the ostium secundum atrial septal defect

**DOI:** 10.5830/CVJA-2013-014

**Published:** 2013-06

**Authors:** Alimohammad Hajizeinali, Mohammad Alidoosti, Hakimeh Sadeghian, Arezoo Zoroufian, Mehrnaz Rezvanfard, Marat A Volman

**Affiliations:** Interventional Cardiology Department, Tehran Heart Centre, Tehran University of Medical Sciences, Tehran, Iran; Interventional Cardiology Department, Tehran Heart Centre, Tehran University of Medical Sciences, Tehran, Iran; Echocardiography Department, Tehran Heart Centre, Tehran University of Medical Sciences, Tehran, Iran; Echocardiography Department, Tehran Heart Centre, Tehran University of Medical Sciences, Tehran, Iran; Research Department, Tehran Heart Centre, Tehran University of Medical Sciences, Tehran, Iran; Department of Medicine, Division of Cardiology, David Geffen School of Medicine at UCLA, Los Angeles, California

**Keywords:** atrial septal defect (ASD), occluder device, 2D TEE, balloon sizing

## Abstract

**Objectives:**

Transcatheter closure of a secundum atrial septal defect (ASD II) has become an effective alternative for surgical treatment. In this study we evaluated the correlation between the two-dimensional transoesophageal echocardiographic (2D TEE) sizing of ASDs and the actual diameter of occluders in patients undergoing device closure.

**Methods:**

The records of 54 patients who underwent transcatheter ASD closure were reviewed. ASD characteristics and maximum defect diameter were evaluated using preprocedure 2D TEE images. Appropriate device size was determined by the balloon sizing method, which measures the balloon occlusive diameter (BOD) via TEE and fluoroscopy. ASD closure was performed under continuous TEE monitoring using the Amplatzer occluder in all patients.

**Results:**

The mean of the TEE-derived maximum defect diameter was significantly lower than the mean of the BOD (17.8 ± 4.5 vs 22.1 ± 5.1 mm; *p* < 0.001) and the mean size of the implanted occluder device (17.8 ± 4.5 vs 23.3 ± 5.1 mm; *p* < 0.001). However, a good correlation was found between the TEE-derived defect size and the BOD (BOD = 0.898 × TEE defect size + 6.212, *R* = 0.824; *p* < 0.001) and between the TEE measurement and the final size of the implanted Amplatzer (device size = 0.928 × TEE defect size + 6.853, *R* = 0.822; *p* < 0.001).

**Conclusions:**

2D TEE may provide a good equation to predict the BOD or the size of the occluder device; however, further studies are needed to investigate whether it is feasible to perform transcatheter ASD occlusion without balloon sizing.

## Abstract

Atrial septal defect (ASD) is one of the most common lesions in congenital heart disease.^1^ The most frequent ASD is of the ostium secundum type (ASD II), which constitutes approximately 7% of all cases of congenital heart disease,^2^ and is suited for transcatheter device closure.^3^,^4^ Percutaneous closure of the ASD II has been accomplished safely and effectively using several different devices.^5^-^7^

Accurate measurement of the size of the defect is of paramount importance for the selection of an appropriate device and its subsequent successful deployment. Implanting too large a device may lead to a mushrooming deformity or cardiac perforation or it may increase the risk of device erosion over time,^8^-^10^ while using too small a device has been accompanied by device instability, distal embolisation of the device, and residual shunting.^11^,^12^ Balloon sizing of the defect has been considered the gold standard for measuring ASD size,^13^-^18^ while angiography, transthoracic echocardiography (TTE), two- and three-dimensional transoesophageal echocardiography (TEE), intracardiac echocardiography, and intravascular ultrasound have been tried as guiding methods during the closure procedure.^10^,^14^-^16^,^18^-^20^

Stretched balloon diameter (SBD) and balloon occlusive diameter (BOD) are two measurements that have long been used by interventionalists in the selection of an appropriate device size for implantation.^17^-^19^ However the balloon sizing method has its disadvantages. Inflation of the balloon may enlarge the defect, cause arrhythmias, or lead to hypotension due to decreased diastolic filling.^21^,^22^ Some investigators therefore consider this cumbersome procedure unnecessary^7^,^23^ and prefer less-invasive measuring methods in the selection of the size of the ASD closure device.^10^,^14^,^19^,^24^

TEE is crucial for the assessment of ASD morphology.^19^,^23^,^25^ Many studies have indicated the highly reliable role of TEE in the prediction of BOD, SBD and device size.^10^,^15^,^17^,^19^

We previously investigated the association between the BOD and pre-procedure TEE-estimated defect size in a study with a smaller sample size.^26^ The main aim of the current study was to compare the ASD II diameter obtained via TEE and the deployed device size, and subsequently devise a formula for estimating the appropriate device diameter using TEE measurement.

## Methods

We retrospectively reviewed the records of patients with ASD II considered for device placement at our institution from July 2005 to February 2010. Of 60 patients, 54 (12 male and 42 female) underwent successful transcatheter closure (device in proper position and no or trivial shunt across the septum) and were included in our study. The procedure failed in four cases due to insufficient support of the device by the interatrial septum, and two other patients underwent open-heart surgery because the device had embolised to the left atrium.

Before the procedure, all the patients underwent a comprehensive transoesophageal echocardiographic study to investigate the morphology of the defect. Based on availability, 36 patients underwent ASD closure using the Amplatzer septal occluder, whereas 18 patients had its Chinese copycat, the Heart® ASD occluder device. Pre-discharge echocardiography was done 24–48 hours after the procedure.

Echocardiographic assessment was conducted in all patients, using a combination of two-dimensional (2D) transthoracic (Vingmed GE, Horten, Norway, 3.5-MHz transducer) and transoesophageal echocardiography (Vivid-7, Vingmed GE, Horten, Norway, 7-MHz transducer). All patients were reassessed between 24 hours and six weeks after PTMC via transthoracic echocardiography. All echocardiographic measurements were assessed based on the American Society of Echocardiography (ASE) guidelines and standards.

TEE was performed within the six-month period before the transcatheter occlusion procedure, to exclude other associated cardiovascular deformities and to investigate the suitability of the ASD size and its surrounding rims for transcatheter closure. Under local anaesthesia, 2D TEE was performed and the diameter of the defect was measured in various planes to determine the maximal defect size. The most useful views for defect sizing included the mid-oesophageal four-chamber view at 0°, the short-axis view at 45–60°, and the bicaval long-axis view at 90–110°.

The maximal diameter of the defect was acquired during the cardiac cycle and recorded. The rims of the defect were measured from the margins of the defect to the inferior vena cava, superior vena cava, right upper pulmonary vein, tricuspid and mitral valves, aorta, and coronary sinus, wherever possible. Exclusion criteria for device closure comprised (1) ASD rims ≤ 5 mm, except for the anterior superior rim, and (2) multiple ASDs as assessed by TEE.

## Balloon sizing and deployment of the septal occluder

Vascular access was obtained from the femoral vein. The tubular sizing balloon (AGA Medical Corporation, Golden Valley, MN, USA) was introduced over a wire that had been placed through the ASD into a left pulmonary vein. Under transoesophageal echocardiographic guidance, the balloon was inflated in the left atrium with increasing quantities of diluted contrast medium and was then pulled back against the ASD. It was thereafter deflated to reach a size sufficient to enable it to be pulled into the right atrium through the defect.

The BOD was defined as the balloon size that completely occluded the ASD and prevented any shunt across the defect without deformity of the balloon. The balloon diameter was measured directly on the screen connected to fluoroscopy and by TEE. Device size was selected with a waist diameter similar to or up to 3 mm larger than the BOD measurement, according to the flexibility of the surrounding rims. Subsequently, the device was inserted and deployed under TEE guidance.

The technique of device closure was similar to those described in the literature.^6^,^27^ Based on availability, the Amplatzer septal occluder (AGA Medical Corporation, Golden Valley, MN, USA) or its Chinese copycat, the Heart® ASD intracardiac patch occluder (Lifetech Scientific INC, Shenzhen, China), were implanted in patients. After releasing the device from the cable, a final TEE examination was undertaken to ascertain the position of the device and any residual shunting.

Successful ASD closure was defined as a device in the proper position with no or trivial leak, as determined by TEE in the catheterisation laboratory. Following the procedure, the patients were sent to a recovery room with ECG monitoring for 12 hours and were discharged two days after the procedure. All patients underwent TTE before discharge.

## Statistical analysis

The numerical variables are presented as mean ± SD (standard deviation), while the categorical variables are summarised by raw numbers and percentages. The paired t-test was used to compare ASD size by TEE and by the diameter of the deployed device or via balloon sizing. The linear regression analysis was performed to demonstrate the relationship between TEE size and BOD and also between TEE measurement and final size of the implanted device.

The measured and calculated (predicted) ASD device diameters were further examined by plotting scattergrams and developing regression lines. The statistical software SPSS version 13.0 for Windows (SPSS Inc., Chicago, IL) was used for the statistical analysis and a *p*-value ≤ 0.05 was considered statistically significant.

## Results

Fifty-four patients (12 male and 42 female) aged nine to 71 years fulfilled the inclusion criteria. All the patients underwent successful ASD closure under 2D TEE monitoring. Maximum defect size ranged between 10 and 30 mm. The devices were deployed appropriately (range of size: 14–39 mm) with no residual shunt across the septum except in one patient who had a trivial shunt just after the occlusion procedure.

Under pre-discharge TTE evaluation, there was no report of any shunt; and mild pericardial effusion occurred in three patients, while moderate pericardial effusion was detected in one patient. In one subject, there was mild compressive effect on the aortic root and in another there was compressive effect on the aortic root, at the base of the anterior mitral leaflet and base of the septal tricuspid leaflet. All these patients were followed up meticulously and these events resolved spontaneously.

**Fig. 1.**
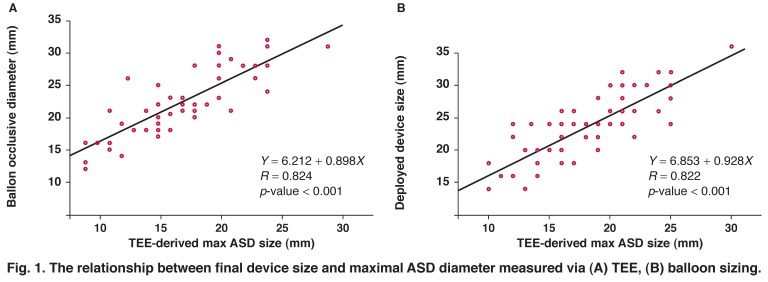
The relationship between final device size and maximal ASD diameter measured via (A) TEE, (B) balloon sizing.

Demographic data, and echocardiographic and ASD characteristics of all patients are summarised in Table 1. The mean of TEE-derived maximum size of the defect was lower than the mean of the BOD (17.8 ± 4.5 vs 22.1 ± 5.1 mm; *p* < 0.001) and also lower than the mean size of the implanted device (17.8 ± 4.5 vs 23.3 ± 5.1 mm; *p* < 0.001) (Table 1). There were good correlations between maximum defect size measured on TEE and via balloon sizing (BOD = 0.898 × TEE defect size + 6.212, *R* = 0.824; *p* < 0.001) and between TEE diameter and the final size of occluded device (device size = 0.928 × TEE defect size + 6.853, *R* = 0.822; *p* < 0.001); Fig. 1 depicts the respective information.

**Table 1.** Demographic, Echocardiographic And ASD Characteristics Of Patients

	**
	
	
	
	
	
	
	
	
	
	
	
	

Data are presented as mean ± SD or *n* (%). TEE: transoesophageal echocardiography; *p < 0.001 compared to the BOD; ^+^*p* < 0.001 compared to the device size.

## Discussion

Our results show that the mean TEE-derived size of the ASD was significantly lower than both the mean diameter of ASD obtained via balloon sizing and the mean size of the implanted device. There was good correlation between TEE sizing of the ASD and the diameter of the deployed device, which makes it feasible to propose a formula that could be used for the prediction of device size in ASD occlusion procedures. However it is not sufficient to predict the exact size of device prior to ASD closure via echocardiographic evaluation.

In line with our results, some reports have previously indicated that TEE underestimates ASD size in comparison with the SBD obtained during catheterisation.^18^,^28^ TEE allows only a limited view of the ASD morphology. The maximum ASD size might therefore be underestimated if the probe is not in the same plane as the largest diameter of the ASD.^18^ It could also be attributed to the intact anatomy of the ASD during echocardiographic evaluation, in contrast with the disturbed anatomy during device implantation, which pushes the atrial walls away.

Accordingly, previous studies have revealed a good linear correlation between TEE-derived ASD size and balloon-sizing measurements,^10^,^15^,^29^ and have also evaluated correlations between TEE measurements and the diameter of the Amplatzer occluder device. They proposed the following equations to calculate proper device size: device size = 2.76 + 1.16 × TEE defect size, *R*^2^ = 0.91;^10^ device size = 4.08 + 1.05 × TEE defect size, *R* = 0.91.^19^

The results of the present study confirm these findings, with some minor differences in the equations. These differences might be attributed to different sample sizes, technical methods, and institution standards. Likewise, several other investigators have confirmed the accuracy of SBD prediction^17^ or occluder device size,^10^,^19^ based on echo measurements.

Some limitations inherent to our study must be taken into account. We only reviewed the records of 54 patients, whose procedures were not performed by a single operator. Consequently, potential differences in the physicians’ experience levels and in the patient population might have been responsible for the minor differences between our formula and the formulae proposed by other investigators.

We excluded multiple ASDs on account of the fact that accurate assessment of the defect diameter in these situations was difficult to obtain. The suitability of TEE for the sizing of multiple defects therefore remains to be elucidated. In addition, the long-term results of transcatheter ASD closure without balloon sizing have not been investigated extensively and await further studies.

## Conclusion

Based on this study, a good formula was developed that correlates TEE measurements of ASD and the size of the implanted device. However further studies are needed to elucidate whether or not this formula alone can be used to replace balloon sizing of ASDs.
